# Deep Learning Image Processing Enables 40% Faster Spinal MR Scans Which Match or Exceed Quality of Standard of Care: A Prospective Multicenter Multireader Study

**DOI:** 10.1007/s00062-021-01121-2

**Published:** 2021-11-30

**Authors:** S. Bash, B. Johnson, W. Gibbs, T. Zhang, A. Shankaranarayanan, L. N. Tanenbaum

**Affiliations:** 1 RadNet—San Fernando Interventional Radiology, 1510 Cotner Ave., 90025 Los Angeles, CA USA; 2 Rayus Radiology, 5775 Wayzata Blvd. Suite 190, 55416 St. Louis Park, MN USA; 3 Dept. of Neuroradiology, Mayo Clinic, 5777 E. Mayo Blvd., 85054 Phoenix, AZ USA; 4 Apple, One Apple Park Way, 95014 Cupertino, CA USA; 5 Subtle Medical, 883 Santa Cruz Ave, 94025 Menlo Park, CA USA; 6 Radnet-Lenox Hill Radiology, 755 Second Avenue, 10017 New York, NY USA

**Keywords:** MRI, Deep learning, Artificial intelligence, Spine, Imaging

## Abstract

**Objective:**

This prospective multicenter multireader study evaluated the performance of 40% scan-time reduced spinal magnetic resonance imaging (MRI) reconstructed with deep learning (DL).

**Methods:**

A total of 61 patients underwent standard of care (SOC) and accelerated (FAST) spine MRI. DL was used to enhance the accelerated set (FAST-DL). Three neuroradiologists were presented with paired side-by-side datasets (666 series). Datasets were blinded and randomized in sequence and left-right display order. Image features were preference rated. Structural similarity index (SSIM) and per pixel L1 was assessed for the image sets pre and post DL-enhancement as a quantitative assessment of image integrity impact.

**Results:**

FAST-DL was qualitatively better than SOC for perceived signal-to-noise ratio (SNR) and artifacts and equivalent for other features. Quantitative SSIM was high, supporting the absence of image corruption by DL processing.

**Conclusion:**

DL enables 40% spine MRI scan time reduction while maintaining diagnostic integrity and image quality with perceived benefits in SNR and artifact reduction, suggesting potential for clinical practice utility.

Deep learning (DL) based image enhancement techniques have gained attention in recent years [1]. DL is a subset of artificial intelligence (AI) machine learning (ML) that uses multiple processing layers to progressively extract key relevant features from the input data. DL models are based on artificial neural networks, most commonly convolutional neural networks (CNN) and variations, in which data transitions through a chain of layers of transformational nodes from input to output, simulating layers of neurons. DL based solutions leverage CNNs to process large volumes of data through a complex framework of decision-making nodes known for exemplary performance in image recognition applications, such as the ability to recognize and categorize image features [2]. DL algorithms are applied to an array of computer vision learning tasks in many industries.

Diagnostic imaging modalities are particularly suited to benefit with opportunities such as reduced radiation and/or contrast dose for PET [3, 4], MR [5] and CT [6]. DL-based image enhancement can boost image signal-to-noise ratio (SNR) offering the potential for reduced scan times [7], enhanced patient experience [8] and improved image center efficiency. DL-based image denoising methods have demonstrated performance advantages over traditional methods of denoising [9, 10] and may be employed to bolster quality of fast acquisition of MR examinations. Fast acquisitions are accomplished by modifying conventional imaging protocol parameters to decrease scan times while maintaining resolution (reducing excitations, raising bandwidth, increasing parallel imaging factors) at the cost of increased image noise (reduced SNR). DL algorithms are then applied to the compromised fast scan data to restore SNR while maintaining image sharpness and standard of care (SOC) image quality.

This prospective multicenter multireader study was designed to evaluate 40% scan time reduced spine MR images processed with a commercially available DL reconstruction algorithm against those obtained with routine SOC scan times. Along with subjective preference rating based on typical imaging criteria, the 3 neuroradiologists also blindly assessed the comparative integrity and consistency of the DL processed images.

To quantitatively assess the integrity of image processing by the DL algorithm, we employed a structural similarity index (SSIM) [11] to evaluate for absolute errors (anatomic or pathologic data loss or aberration), and per pixel L1 difference to evaluate for differences in signal intensity.

## Material and Methods

### Participants

A total of 61 consecutive patients (45.5 ± 17.1 years old) were prospectively recruited and consented for this multicenter, multireader, randomized case-control Institutional Review Board (IRB) approved study. Each patient (28 females, 33 males) was scheduled to have a clinically indicated MRI of the cervical, thoracic, or lumbar spine.

### Image Acquisition

MR imaging consisted of 14 cervical, 9 thoracic and 88 lumbar region image sets (*n* = 111). The studies were acquired from one of 5 scanners (GE; Waukesha, WI, USA, 1.5 T HDe, 2 GE 1.5 T HDxt, Siemens; Erlangen, Germany, 3 T Skyra, Siemens 3 T Verio) at 5 imaging centers (4 New York, 1 California). A clinical practice, SOC study was performed consisting of multiple routine pulse sequences: sagittal T2 (*n* = 23)/T1 (*n* = 21)/STIR (*n* = 18)/PD (*n* = 12); and axial T2 (*n* = 20)/T1 (*n* = 17) (average sequence scan time: 171.2 ± 66.4 s) for a total of 111 sequences acquired from 61 patients. In addition, each subject underwent matched pulse sequences with an accelerated (FAST) protocol (average sequence scan time: 96.2 ± 41.2 s), for an additional 111 sequences (Table 1).

**Table 1:** Protocol Parameters. Typical scanning parameters for standard-of-care (SOC) and accelerated (FAST) acquisitions at 1.5 T

	Scan Time	TR	TE/TI	Slice (mm)	Matrix Size	ETL	NEX
*Sag T1*							
SOC	2:57	1550	10	4	320 × 192	8	4
FAST	1:32	1550	10	4	320 × 192	8	2
*Sag T2*							
SOC	1:31	2584	110	4	320 × 224	21	2
FAST	0:36	2584	110	4	320 × 224	21	1
*Sag PD*							
SOC	1:25	1367	35	4	320 × 224	8	2
FAST	0:47	1367	35	4	320 × 224	8	1
*Sag STIR*							
SOC	2:01	4850	33/140	4	320 × 224	8	2
FAST	1:13	4850	33/140	4	320 × 224	8	1
*Ax T2*							
SOC	1:38	4459	102	4	320 × 224	32	3
FAST	0:30	4459	102	4	320 × 224	32	2
*Ax T1*							
SOC	3:15	634	13	4	320 × 224	3	2
FAST	1:47	634	13	4	320 × 224	3	1

### Image Processing

The DL model was trained on 1000s of MR DICOM datasets from multiple vendors and clinical sites with a variety of clinical indications and field strengths, thus experiencing a range of image quality, tissue contrasts, acquisition parameters, and patient anatomies. DICOM-based processing does not utilize proprietary raw k‑space input and is thus vendor agnostic. DL processing provides structure-preserving noise reduction, and the spine model does not remove imaging artifacts or intrinsically enhance image sharpness.

The DL algorithm implements image enhancement using convolutional neural network-based filtering. Original images are enhanced by running through a cascade of filter banks, where thresholding and scaling operations are applied. Separate neural network-based filters are obtained for noise reduction. The parameters of the filters were obtained through an image-guided optimization process [12–14].

The model training process typically involves several steps:

- Initialization: initialize filters and weights with small random values (e.g., random Gaussian weights).
- Forward propagation: provide training images as input to the network, propagate them through the various operations (convolution, rectified linear unit, maximum pooling, etc.), and compute the network output.
- Error calculation: calculate the errors in the output layer (target image vs. output image). Usually a final loss function (for example, sum-of-squared-error) is used to combine the error in each pixel into a single objective value which is (ideally) minimized during model training.
- Back propagation: calculate the error loss gradients with respect to all weights in the network and use techniques like gradient descent to update all filter values/weights and parameter values to minimize the output error/loss.
- Training: repeat the previous steps with all the images in the selected training dataset (e.g., 90% of available dataset), which is called one epoch. Usually multiple (such as 100) epochs are used in model training to optimize/minimize the error objective function (described in step #3) until the model converges into a stable result.

DL processing of the FAST scan data set (FAST-DL) was performed on an edge positioned HIPAA compliant server-virtual machine using an FDA-cleared deep learning-CNN based, image enhancement product, SubtleMR™ (Version 1.2, Subtle Medical, Menlo Park, CA, USA) with a processing time of approximately 30 s per series. All images were reviewed on a commercial DICOM viewer.

### Radiologic Assessment

Three experienced neuroradiologists (>17 years of experience) were presented with 666 different image series randomized in sequential and left-right display order from SOC (*n* = 111), FAST (*n* = 111), FAST-DL (*n* = 111) as paired side-by-side datasets (SOC vs. FAST, SOC vs. FAST-DL, FAST-DL vs. FAST). Image features were preference rated on a Likert scale (1–5), with 1 indicating pronounced perceived superiority of the image on the left, 2 mildly superior on the left, 3 no difference, 4 mildly superior on the right, and 5 significantly superior on the right for (1) perceived SNR; (2) perceived spatial resolution; (3) imaging artifacts; (4) cord delineation; (5) cord/CSF contrast; (6) disc related pathology; (7) bone lesions; and (8) facet/ligamentous pathology. Additionally, all 3 readers assessed each of the paired datasets for image consistency (presence or absence of apparent loss, corruption, alteration, creation or exaggeration of observed anatomy and pathology).

### Statistical Analysis

Wilcoxon rank sum tests were performed to assess the statistical significance of the difference in scores for each feature in comparative datasets (Table 2). Statistical significance of the difference in scores of a dataset feature was determined by a *p*-value <0.05. Mean and standard deviations for the combined reader Likert scores for each feature were also calculated.

**Table 2:** Wilcoxon rank sum test results. All readers combined. *P*-value <0.05 (bold) suggests statistical significance for features in one dataset with respect to its comparison

Feature	SOC vs. FAST		FAST-DL vs. SOC		FAST-DL vs. FAST	
	Mean ± Std	*P* value	Mean ± Std	*P* value	Mean ± Std	*P* value
SNR	3.7 ± 0.5	**<0.05**	3.4 ± 0.6	**<0.05**	3.9 ± 0.4	**<0.05**
Resolution	3.0 ± 0.3	**<0.05**	3.0 ± 0.3	0.41	3.0 ± 0.2	0.25
Artifacts	3.3 ± 0.6	**<0.05**	3.1 ± 0.5	**<0.05**	3.1 ± 0.3	**<0.05**
Cord delineation	3.1 ± 0.2	**<0.05**	3.0 ± 0.2	0.25	3.0 ± 0.1	0.16
Cord/CSF contrast	3.1 ± 0.3	**<0.05**	3.0 ± 0.3	0.56	3.0 ± 0.1	1
Disc pathology	3.1 ± 0.2	**<0.05**	3.0 ± 0.2	0.64	3.0 ± 0.1	0.1
Bone lesions	3.1 ± 0.3	**<0.05**	3.0 ± 0.3	0.3	3.0 ± 0.1	0.41
Facet/ligamentous pathology	3.1 ± 0.2	**<0.05**	3.0 ± 0.1	0.26	3.0 ± 0.1	**<0.05**

Inter-reader agreement was assessed using the Spearman rank correlation method. The coefficient varies from −1 to 1, with −1 indicating a perfectly negative relationship (a high rating from one neuroradiologist and low rating from another) and 1 indicating a perfectly positive relationship (Table 3).

**Table 3:** Spearman Rank-order correlation coefficient for inter-reader agreement. The scores were averaged across the reader pairs. The results indicate moderately strong inter-reader agreement for Likert scale analysis of across all 8 quality features assessed

Spearman Rho	Radiologist 1 vs. 2	Radiologist 1 vs. 3	Radiologist 2 vs. 3
	Rho = 0.454	Rho = 0.527	Rho = 0.442

To quantitatively assess the integrity of images processed by the DL algorithm, we compared both FAST and FAST-DL images to the reference SOC image. We employed SSIM to assess for absolute errors (anatomic or pathologic data loss or aberration), and per pixel L1 difference to evaluate differences in signal intensity. In addition, while not part of the subjective analysis, SOC images were also processed with DL and subjected to SSIM measures (SOC vs. SOC-DL) as an additional method of assessing the impact of DL processing (Table 4).

**Table 4:** Structural similarity index (*SSIM*) results. Quantitative assessment of image similarity using the SSIM was 0.981 ± 0.011 for SOC vs. SOC-DL and 0.984 ± 0.009 for FAST vs. FAST-DL. This supports the absence of substantial anatomic aberration by DL-processing of the source series

Structural Similarity Index (SSIM)	
SOC vs. SOC-DL	0.981 ± 0.011
FAST vs. FAST-DL	0.984 ± 0.009

## Results

### Performance

All 666 image sets (SOC, FAST, FAST-DL) were ranked as of diagnostic quality by each of the 3 neuroradiologists.

FAST-DL was statistically better than SOC for perceived SNR (3.4 ± 0.6, *p*-value <0.05) and imaging artifacts (3.1 ± 0.5, *p*-value <0.05). FAST-DL and SOC were statistically equivalent for perceived spatial resolution (3.0 ± 0.3), cord delineation (3.0 ± 0.2), cord/CSF contrast (3.0 ± 0.3), disc-related pathology (3.0 ± 0.2), bone lesions (3.0 ± 0.3) and facet/ligamentous pathology (3.0 ± 0.1) (with *p*-values >0.24). SOC was better than FAST for all criteria (*p*-value <0.05). FAST-DL was better than FAST for SNR, artifacts, and facet/ligamentous pathology (*p*-value <0.05). Wilcoxon Rank Sum test results for Likert scale analysis are collectively summarized for all 3 readers in Table 2.

**Fig. 1:**
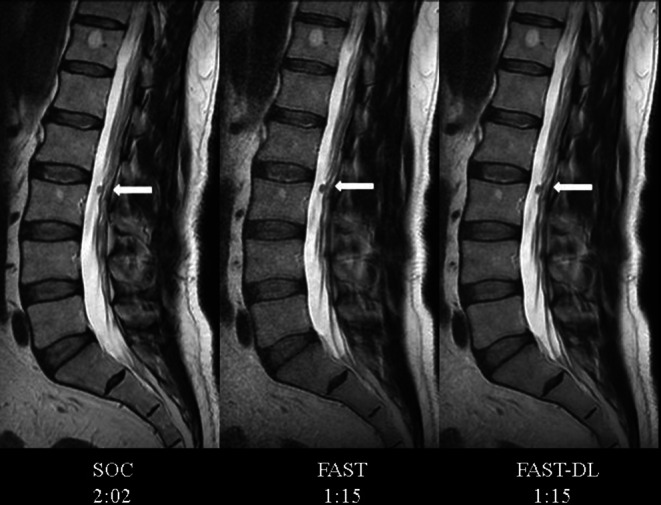
Consistency across datasets. Sagittal T2 (*left to right*): SOC, FAST, FAST-DL with acquisition times. Blinded readers found no variations in image integrity (morphology/pathology) across the datasets. A tiny incidental intrathecal schwannoma (*white arrow*) at upper L3 level maintains excellent visual conspicuity across all three datasets

**Fig. 2:**
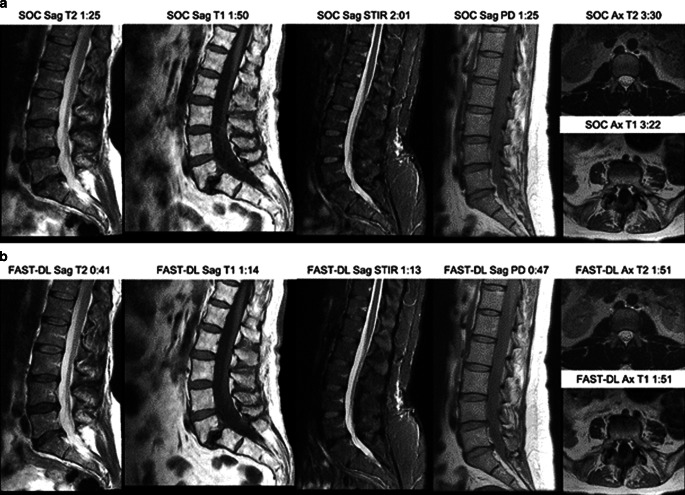
Multisequence imaging. SOC (**a**) and FAST-DL (**b**) Representative patients and acquisition times

Qualitative assessment of image integrity was equivalent across the 3 datasets for all 3 blinded readers, indicating that there was no perceived loss or aberration of anatomy or pathology (Fig. 1). Multisequence imaging of SOC and FAST-DL of representative patients and acquisition times are demonstrated in Fig. 2.

Spearman rank-order correlation coefficient analysis demonstrated moderately strong inter-rater agreement between the 3 blinded neuroradiologists (Rho = 0.454 for radiologist 1 vs. 2; Rho = 0.527 for radiologist 1 vs. 3; and Rho = 0.442 for radiologist 2 vs. 3) (Table 3).

Quantitative assessment of image similarity using the SSIM was 0.981 ± 0.011 for SOC vs. SOC-DL and 0.984 ± 0.009 for FAST vs. FAST-DL. This supports the absence of substantial anatomic aberration by DL processing of the source series (Table 4). The per pixel L1 difference for SOC vs. FAST was 37.5 ± 17.6, and for SOC vs. FAST-DL was 36.7 ± 17.4. The Wilcox paired test differences were found to be significant (*p* < 0.001) at 4.363 e^−12^.

## Discussion

This prospective, randomized, multicenter study assessed the ability of DL enhancement to preserve perceived MR spine image quality despite 40% scan time reduction.

Blinded assessments by 3 neuroradiologists found the overall diagnostic quality of DL-enhanced MR images statistically equivalent or subjectively better than SOC across all assessed features.

MR image quality and speed are traditionally linked by constraints over signal-to-noise ratio. Scans with higher SNR and/or spatial resolution are perceived as offering better overall image quality and greater detail but requiring longer scan times when using traditional image reconstruction techniques. DL-based models in image reconstruction can overcome the SNR/scan time relationship by applying detail-preserving denoising to accelerated sequences and restoring quality to SOC levels. In our study the DL-enhanced fast images were able to provide perceived SNR benefits over even conventional SOC imaging.

MR examinations are susceptible to image degradation from artifacts, often due to patient motion related to long scan times. Motion is a significant challenge in MRI occurring in 29% of inpatient/emergency department examinations and 7% of outpatient studies [15] and can lead to the need to have to repeat portions of or even complete examinations. Andre et al. found that that 19.8% of all MRI sequences need to be repeated due to motion artifact, which extrapolates to a $ 592 revenue loss per hour and $ 115,000 loss annually per scanner due to motion artifact [16]. In this study, DL-enhanced images statistically exceeded SOC in artifact reduction, likely reflecting shorter scan times and reduced patient motion.

Scan time reductions inherently improve patient comfort and overall experience [8]. Up to 30% of patients reported significant anxiety, largely from claustrophobia, during an MR study [17]. The authors’ internal multicenter surveys have shown that even minor reductions in examination length result in a significantly higher level of patient satisfaction [8].

In our study, we achieved a scan time reduction of approximately 40% while maintaining or exceeding routine quality. If DL-enhanced fast protocols were utilized with all MR exams, one could anticipate a proportional increase in exam-based workflow efficiency for an imaging facility. Future research could explore whether scan time reduction of this scale results in a true positive impact on profitability, e.g., the ability to scan more patients per day.

A scan time acceleration of 40% was chosen for this study based on limited clinical experience. Future research might investigate greater accelerations. Work with the brain has shown image acceleration of 60% while maintaining quantitative integrity [18]. Additional research could focus on making greater image quality practical by denoising higher resolution native acquisitions.

In this study, the SOC images serve as the standard for image preference. Our randomized blinded assessment of the imaging features is meant to reflect human visual perception of comparative image quality. A radiologist’s qualitative assessment of non-inferiority is critical before a DL-enhanced alternative would be considered acceptable for clinical use. On the other hand, processed images should satisfy both qualitative and quantitative measures to ensure that diagnostically relevant features are not altered, and integrity of the processed image information is maintained.

Concerns exist about DL post-processing introducing instabilities in an image, where tiny perturbations in the sampling domain have been shown to be capable of translating into noticeable artifacts on the reconstructed image [19]. This has been shown for highly contrived noise additions to k‑space data and it is unclear whether such effects occur under normal operating conditions. It is important to emphasize that the current method starts from image-based data rather than k‑space, which may be less susceptible to this effect.

However, to verify the absence of data aberration on the DL post-processed images, the quantitative metric of SSIM [11] was calculated to assess for the presence or absence of absolute errors (such as anatomic data loss or exaggeration) for the pairs of accelerated unenhanced and DL-enhanced datasets (FAST vs. FAST-DL), and as an additional measure, for the SOC series and one processed with DL solely for this purpose (SOC vs. SOC-DL). While SSIM has limitations [20], it is a commonly employed metric to measure the similarity between two images, ranging from 0.0 to 1.0, with 1.0 meaning two images are identical. The high SSIM results for FAST vs. FAST-DL and SOC vs. SOC-DL are reassuring with respect to the absence of significant DL-processing related corruption. As the SOC and FAST scans represent two separate acquisitions with minor differences in patient and slice position, SSIM for these could not be accurately assessed. As an additional quantitative assessment of image similarity, L1 measures were obtained. The quantitative result for image integrity is consistent with the blinded qualitative assessment by the 3 neuroradiologists who reported no instances of observed image aberration between dataset pairs (Fig. 1).

While there are numerous AI-centric solutions in the medical imaging marketspace, many have narrow application. The broad benefits of a DL solution for cross-sectional image reconstruction have been recognized, and at present MR and CT manufacturers are developing or refining DL solutions for image processing, currently at variable stages of fruition and regulatory clearance [21, 22]. Scanner vendors will likely limit their proprietary DL solutions to their own devices, and at least initially, to their newest high-end scanners [20, 21]. Independent or third-party DL solutions are vendor-agnostic and model-neutral, increasing appeal to imaging enterprises operating scanners with a variety of vendors, models, and ages.

The generalizability of our findings could be strengthened by further investigations and larger subject populations, given the relatively small number of uncommon pathologies within this study cohort. Of note, only a single intradural lesion was present and no intramedullary lesions were detected in this outpatient study and thus reported measures of cord delineation and cord/CSF contrast therefore serve as surrogates for evaluation of intradural pathology. Pathologies commonly present on outpatient spine MR studies, such as disc derangements, spinal canal stenosis, and facet arthropathy were well represented and faithfully preserved across all three datasets (SOC, FAST and FAST-DL).

In this study, clinical spine imaging patients were enrolled in a consecutive manner, a method which could both reduce as well as create bias. This led to a disproportionate number of lumbar spine studies with respect to cervical and thoracic exams; however, at the time of statistical analysis, the blinded Likert rating trends, such as perceived benefits in SNR and artifact reduction, were found to be equally applicable across all spine exams regardless of the anatomic target location.

Strengths of this investigation include the prospective, multicenter, multireader study design with images obtained from geographically diverse patient populations, using magnets of variable strength, age, and manufacturer. The results, despite evaluation in limited number of patients, support the feasibility and suggest the generalizability of DL enhancement to shorten clinical MR spine examinations.

## Conclusion

DL matches or exceeds the perceived image quality and diagnostic qualitative performance of standard of care spine MRI exams, enabling a 40% scan time reduction. DL qualitatively outperformed standard of care in reduction of image artifacts and perceived signal-to-noise ratio. Quantitative structural similarity index metrics (SSIM) attest to image integrity preservation after DL-processing. This study suggests the potential for routine utility of DL reconstructed MRI in clinical practice.

## Abbreviations

AI: Artificial intelligence
CNN: Convolutional neural network
DL: Deep learning
ML: Machine learning
NEX: Number of excitations
SNR: Signal-to-noise ratio
SOC: Standard of care
SSIM: Structural similarity index measure
